# Phase coexistence in the fully heterogeneous Hegselmann–Krause opinion dynamics model

**DOI:** 10.1038/s41598-023-50463-z

**Published:** 2024-01-02

**Authors:** Rémi Perrier, Hendrik Schawe, Laura Hernández

**Affiliations:** Laboratoire de Physique Théorique et Modélisation, UMR-8089 CNRS, CY Cergy Paris Université, Cergy-Pontoise, France

**Keywords:** Physics, Statistical physics, thermodynamics and nonlinear dynamics, Complex networks

## Abstract

We present an extensive study of the joint effects of heterogeneous social agents and their heterogeneous social links in a bounded confidence opinion dynamics model. The full phase diagram of the model is explored for two different network’s topologies and compared to two opposed extreme cases: on one hand, the heterogeneous agents constitute a mixed population and on the other, their interactions are modeled by a lattice. The results show that when agents prone to compromise coexist with close-minded ones, the steady state of the dynamics shows coexistent phases. In particular, unlike the case of homogeneous agents in networks, or heterogeneous agents in a fully mixed population, it is possible that the society ends up in consensus around one extreme opinion. Moreover, during the dynamics, the consensus may be overturned from one extreme to the other of the opinion space. We also show that the standard order parameter, the normalized average size of the largest opinion cluster, may be misleading in this case, as it hides the existence of these phases. The phase where the opinion of the society is overturned does not require the presence of agents with special characteristics, (stubborn, extremists, etc.); it results from the interplay of agents which have agreed on an extreme opinion with the remaining group that holds the opposite one. Among the former, some may be prone to compromise with other agents which are out of the majority group, these agents, according to their location in the network, may act like bridges between the two groups and slowly attract the whole society to the other extreme.

## Introduction

Nowadays, the challenge of theoretical studies on opinion dynamics is to consider realistic properties that were absent from the first stylized models in order to explore their role on the outcomes of the dynamics^1,2^. These original stylized models have nevertheless been successful in revealing the leading role played by the microscopic interactions at the level of the agents, often inspired by Social Influence Theory^3,4^, on the transition from a fragmented opinion state to another were polarization around two different opinions or full consensus is observed. Amongst the most famous results, one can cite the low threshold for consensus in bounded confidence models^5,6^, the intrinsic dynamical character of segregation^7^, or the unexpected coexistence of multicultural societies, in spite of the presence of local homogenizing interactions^8^.

The ultimate goal in this domain would be to fill the gap between the numerous theoretical models and the growing number of empirical studies derived from the increasing amount of data that is nowadays easily accessible, mainly due to the widespread usage of social networks. However, a model that integrates as many details of a real society as possible will not be explanatory, due to the large number of uncontrolled parameters. Instead, the safe road to the comprehension of the behaviour of realistic models is to systematically study the role that each of the main properties characterizing real social systems, play on the outcomes of the dynamics.

An unavoidable aspect to be considered in the study of real social systems is heterogeneity. Social actors and their interactions are intrinsically heterogeneous, displaying idiosyncratic characteristics that may evolve -when they do- at a very slow timescale with respect to that of the opinion formation. On this line, several works have extended existing models to include some realistic properties of social interactions. An important extension takes into account that not all the agents interact with the same number of people and certainly not with the whole population, as in the fully mixed population hypothesis, or *mean-field approach*; instead, social interactions are modeled by a network of social agents connected by links which may be endowed with the properties of social ties observed in real life^9–11^.

Another source of heterogeneity is rooted in the fact each individual has personal characteristics that lead to different reactions to the same stimuli. This is usually modeled by an initial distribution of random variables representing these idiosyncratic properties over the population, which remain fixed during the dynamics of opinion formation. In Physics this situation is known as *quenched disorder* and its introduction generally leads to important modifications of the results established for models without disorder. This is the case also for opinion dynamics models, even in the simple mixed population scenario^12–17^.

The full phase diagram of the heterogeneous version of the Hegselmann–Krause (HK) opinion dynamics model^6^ in a mixed population setting has recently been extensively studied^16^. The *bounded confidence* HK model is based on two main ingredients of social interaction, *homophily*, which designates the fact that “likes attract” and *social influence*, which correspond to the fact that humans have the tendency to be influenced by those who are similar to them, while they disregard the opinions of others whose opinion is far from their own.

In the HK model, opinions are allowed to vary continuously, and therefore a continuous dynamical variable is associated to each agent representing the evolution of its opinion in time. Social agents interact only with those peers whose opinion differ from their own in less than a parameter value, $\varepsilon$, known as *confidence*, materializing the open-mindedness of the society. At a difference with the Deffuant model, which considers asynchronous pairwise interactions^5^, in the HK model the dynamics is a synchronous operation, where all the agents update their opinion to the average opinion of the set of agents they interact with, and their own. The corresponding heterogeneous version considers that, instead of having a single confidence value for the whole population, the confidence of social actors is an idiosyncratic characteristic: while some are very *open-minded*, ready to interact even with unlike peers, others are *close-minded* and interact only with those holding an opinion very similar to theirs. As it is not trivial to parameterize the open-mindedness with real data, a whole exploration of the phase diagram is necessary. The main result is that, paradoxically, introducing additional more open-minded agents in a society that contains some close-minded ones, does not necessarily lead to a consensus phase, i.e. a final state where all the agents of the society converge to the same opinion.

On the other hand, it has been shown by extensive finite size analysis, that when the agents are homogeneous (all holding the same value of the confidence), the introduction of heterogeneity in the social ties, constraining them by a random network, makes the critical value of the confidence that allows to establish consensus, $\varepsilon _c$, decrease with the system size, vanishing for $N\longrightarrow \infty$ (thermodynamic limit). This effect is absent in fully mixed populations and lattices^11^.

Real societies mix these two sources of heterogeneity, as they are composed of heterogeneous agents who are linked by a network of social contacts that limits the possibilities of social interaction. In this work we present a new step towards the understanding of real systems, by integrating both aspects in a single model: heterogeneous agents with heterogeneous connections. To do so, we consider a HK model where each agent, *i*, has its own value of confidence $\varepsilon _i$ and where their potential interactions are constrained by a network. Networks of different topology are studied including regular, spatially embedded ones (lattices) and the complete network setting. Our results show that the combination of both types of heterogeneity gives rise to non trivial phenomena; in particular, the presence of coexisting *phases*, understood as global opinion states, where the society shows either a weak consensus around the middle opinion (i.e. $x \approx 0.5$), or where a strong consensus around an extremist opinion (i.e. $x \approx 1$ or $x \approx 0$ ) is observed. This work also reveals that a few open-minded agents may be able to overturn the majoritarian opinion from one extreme to the other. By comparing with previous results, we identify the role of each aspect of heterogeneity in the observed outcomes of the dynamics, and we reveal new phenomena that are the result of their joint action.

## Results

We have performed extensive simulations of the heterogeneous HK model where the connections among the agents are constrained by different types of networks.

Each society is characterized by a set of variables representing the confidence of each agent *i*, $\varepsilon _i$, uniformly distributed in the interval $[\varepsilon _l,\varepsilon _u]$, representing the range of open-mindedness of the society. The confidences are fixed in time as they represent an idiosyncratic property of the agents. This is the equivalent of a *quenched disorder* in physical systems. Moreover, social ties are modeled by a fixed network, where the vertices represent the agents and the links restrict their possible interactions to their neighbours in the network.

The opinion of each agent *i* at discrete time *t* is represented by the *dynamic* variable $x_i(t) \in [0,1]$. Each agent *i* interacts with its neighbours in the network, provided that their difference of opinion is smaller than $\varepsilon _i$. It should be noted that the heterogeneity in the confidence values introduces an asymmetry in the interaction with respect to the homogeneous version of the model^6,9–11^ (see Methods).

For each simulation, the agents’ initial opinions $x_i(t=0)$ are uniformly distributed in the interval [0, 1]. Unless stated otherwise, averages are computed over 1000 samples of each society, except for the phase diagrams, where due to the detailed covering of the phase space, averaging was limited to 100 samples for the networked systems.

### Phase diagram of the model considering different network topologies

**Figure 1:**
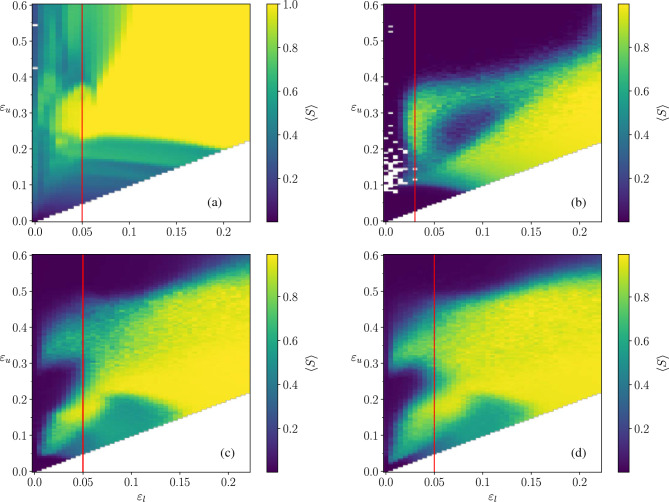
Phase diagram for the heterogeneous HK model in systems of $N=16384$ agents. The color map represents the average normalized size of the largest cluster $\langle S \rangle$ at the steady state of the system. (**a**) Fully connected network (taken with the permission of the authors) from^16^. This depicts the average over 1000 realizations for each of the 2923 points, with a resolution of $6.25\times 10^{-3}$ on $\varepsilon _l$ and $\varepsilon _u$. (**b**) Square lattice including up to third neighbour interactions, so that the average degree is $\langle k \rangle = k = 12$. (**c**) Erdős–Rényi network with $\langle k \rangle =10$ and (**d**) Barabási–Albert network with $\langle k \rangle =10$. Panels (**b**–**d**) show an extensive exploration of the phase space with 4455 different points, with a resolution of $5\times 10^{-3}$ on $\varepsilon _l$ and $\varepsilon _u$. In this case, averages have been taken over 100 realizations. White pixels correspond to societies where we did not compute the average to avoid selection bias because of a few realizations which did not converge in reasonable computing time (see section “Particular case: lattices” below). Red lines denote the $\varepsilon _l = 0.05$ and $\varepsilon _l = 0.03$ slices to be discussed in detail later.

The color map of Fig. 1 represents the phase diagram of the system based on a standard order parameter, usually considered in opinion dynamics studies: the average normalized size of the largest opinion cluster at the steady state, $\langle S \rangle$, of the HK dynamics for different societies. Each panel corresponds to a different topology of the interactions’ network and, inside each panel, each pixel corresponds to $\langle S \rangle$, calculated by averaging over 100 realizations of a given society. A given society is defined by the coordinates $(\varepsilon _l, \varepsilon _u)$ giving the interval from which the confidence parameters of the agents have been drawn, and by type of network representing their potential interactions. Clearly, the resulting phase diagram strongly depends on the interplay of the heterogeneity of the agents and the topology of the network that constrains the possible interactions.

Figure 1a, corresponds to the case of the mixed population studied in Ref.^16^ and is given as a reference. The effects exclusively related to the network constraining the interactions appear along the diagonals which represent homogeneous systems: the critical value to reach consensus, $\varepsilon _c$, diminishes in all networked societies with respect to the fully mixed population^11^. On the other hand, the effect of heterogeneity is also observed in all the panels: in societies with low confidence agents (low $\varepsilon _l$), adding more confident agents does not always enhance consensus. The strongest effect of combining both properties (heterogeneous agents and networked societies) can be observed along the red vertical lines in Fig. 1: the order parameter $\langle S \rangle$ falls down to zero in networked societies where polarization was observed in the mixed population. (See Supplementary material for the phase plots of lattices of different coordination numbers. A 360$^{\circ }$ visualization of the 3D scatter plots of the largest cluster size for each realization in the steady state, leading to the average represented in Fig. 1, can be found in the Supplementary Material I.A.)

In order to understand the differences with the mixed population case, we study in detail systems located in the region where a non monotonous behaviour was found in Ref.^16^, marked with a vertical red line in Fig. 1.

**Figure 2:**
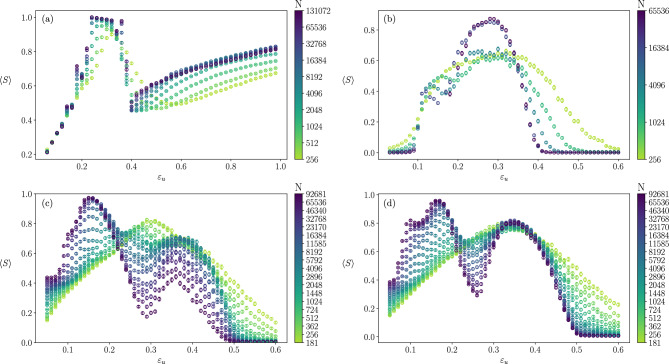
Average relative size of the largest cluster, $\langle S \rangle$, over 1000 realizations as a function of $\varepsilon _u$ for fixed $\varepsilon _l$ corresponding to the red vertical line of Fig. 1. The color map indicates different considered system sizes. (**a**) Fully connected network (data taken with the permission of the authors from^16^), $\varepsilon _l=0.05$. (**b**) Square lattice including up to third neighbour interactions, $\varepsilon _l=0.03$. (**c**) Erdős–Rényi network of $\langle k \rangle =10$, $\varepsilon _l=0.05$. and (**d**) Barabási–Albert network of $\langle k \rangle =10$, $\varepsilon _l=0.05$.

Figure 2 shows the order parameter, $\langle S \rangle$, as a function of $\varepsilon _u$, for fixed $\varepsilon _l$, different system sizes, and different networks’ topology (the behaviour of other quantities, is shown in the Supplementary Material). Strong size effects are observed and, although the global shape of the curves seems to stabilize as *N* increases, the non monotonous behaviour of the order parameter prevents us from finding a single scaling form. A similar qualitative behaviour is observed for both random networks except for the behaviour of the second peak with *N*.

**Figure 3:**
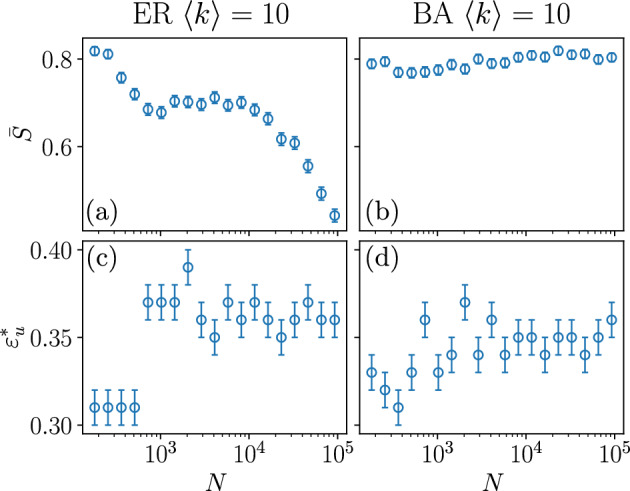
Comparison of size effects on the second peak of the order parameter, $\langle S \rangle$, as a function of the system size for ER and BA networks with $\varepsilon _l = 0.05$. (**a**,**b**) ${\bar{S}}$, the height of the second peak, defined as the maximum value reached by $\langle S \rangle$ in the interval $\varepsilon _u\in [0.3, 0.5]$, (**c**,**d**) $\varepsilon _u^*$, position of the second peak.

Figure 3 compares the scaling behaviour of the location and the height of this second peak, showing that it vanishes for large *N* for the ER network, while it stays for the BA topology. In both cases, the position of the second peak does not appear to shift with the system size and stabilises around $\varepsilon _u = 0.36$ and $\varepsilon _u = 0.35$ respectively.

### The coexisting phases

The previous results show that, unlike the mixed population case, when the potential interactions are constrained by a network, $\langle S \rangle \longrightarrow 0$ for large values of $\varepsilon _u$, which seems counter-intuitive. In Ref.^16^ it was shown that, for low values of $\varepsilon _l$, as the fraction of open-minded agents increases, consensus turns into polarisation. This is caused by the fast convergence of the very open-minded agents to a central opinion, which leaves aside the close-minded ones. The latter, becoming unable to interact with the majoritarian strand, form a secondary cluster of relative importance, leading to a polarized opinion state. Here, this explanation does not hold as there is no dominant cluster. In other words: the open-minded ones do not form a large cluster either.

**Figure 4:**
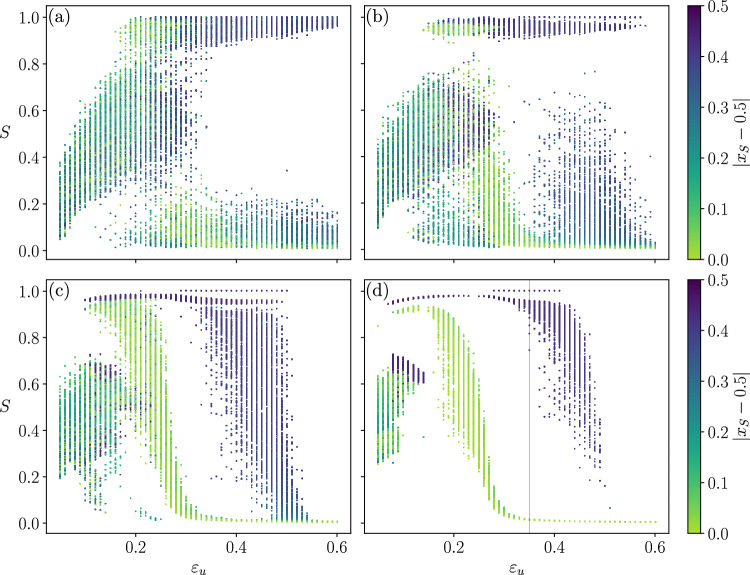
Detail of size effects for the HK model in the Erdős–Rényi network of $\langle k \rangle =10$. Results for 1000 samples, $\varepsilon _l=0.05$. (**a**) $N=256$, (**b**) $N=1024$, (**c**) $N=8192$, (**d**) $N=92{,}681$. Red line marks the value $\varepsilon _u=0.35$.

In order to clarify this point it is necessary to examine the distribution of largest clusters over all the realizations. In Fig. 4 we show, for different system sizes, *N*, the scatter plot of the largest clusters’ normalized sizes, *S*, for 1000 realisations of the same society (same network, confidences drawn from the same interval, $[\varepsilon _l=0.05,\varepsilon _u]$), in the steady state. The color code represents the *extremism*, *e*, of the opinion held by the members of the largest cluster of each realization, i.e. its shift from the central opinion, which corresponds to the steady state for consensus in the homogeneous system ($x_S(t \longrightarrow \infty )=0.5$), where $x_S$ denotes the opinion of the members of the largest cluster. Finite size effects are very strong, changing qualitatively the structure of the scatter plots which stabilizes only at large *N*, revealing the presence of coexistent phases. Although finite size effects are well known in equilibrium thermodynamics, the results shown here, point out again at the necessity of considering large enough sizes so that the observed properties stabilize, which is unfortunately, still too often overlooked. Notice that in panel (b), corresponding to $N=1024$, a typical size studied in the literature, the structure is very different from the stable one. Similar finite size effects are observed for the other studied networks (see Supplementary Material sections II.A and II.B for videos of the evolution of the scatter plots due to finite size effect, for ER and BA networks).

Focusing on the largest studied size, $N=92681$, shown in Fig. 4d, it is clear that the dispersion of *S* as well as the corresponding opinions strongly depend on the value of $\varepsilon _u$. In particular, for intermediate values of $\varepsilon _u$, like the one marked by a red line in Fig. 4d, societies where the largest cluster converges to a mild opinion ($x_S (t \longrightarrow \infty ) \approx 0.5$) coexist with others where the largest cluster holds an extremist opinion (either $x_S (t \longrightarrow \infty ) \approx 0$ or $x_S (t \longrightarrow \infty ) \approx 1$). We will see that the former do not form consensus at a given opinion but are constituted of a large number of agents holding *different opinions* around the central one. This explains the very low values of S in the scatter plot leading to the collapse of the value of $\langle S \rangle$, observed in Fig. 2. This implies that when the interaction among heterogeneous agents is constrained by a network, $\langle S \rangle$ is not a suitable order parameter. When the network of possible interactions has the Barabási–Albert topology, the same general qualitative behaviour is observed. However, as mentioned above, the peak of the order parameter at $\varepsilon _u^* \approx 0.35$, which diminishes with the size in Erdös–Renyi networks Fig. 2a, stays for Barabási–Albert networks Fig. 2b, as shown in Fig. 3.

**Figure 5:**
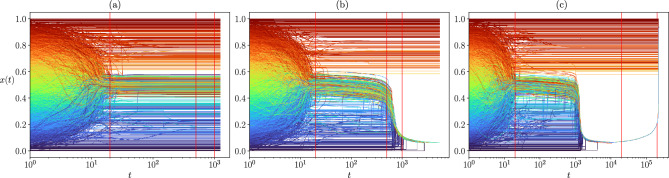
Examples of the opinion evolution of the agents for a society where the interactions are constrained by an Erdős–Rényi network of $\langle k \rangle = 10$, and with confidence interval $\varepsilon _l = 0.05$, $\varepsilon _u = 0.35$, $N=92681$. The vertical lines indicate the times corresponding to the snapshots shown in Fig. 7. (**a**) *Mild* phase: The agents located around the central opinion do not meet the criterion ($\delta x < 10^{-4}$ ) to constitute a single cluster. (**b**) *Skewed* phase: The final state is a dense strand containing the majority of the society while a few agents remain isolated. (**c**) *U-turn phase*: The opinion of the society is overturned from one extreme to the opposite one, the whole society is involved. Note that for readability and computational cost, only the evolution of 4000 agents taken at random is displayed.

In order to understand the nature of the coexistent phases, we have studied individual trajectories corresponding to each of them. Figure 5 shows three examples of trajectories, one in each of the observable phases at the point (0.05, 0.35) of the ($\varepsilon _l, \varepsilon _u)$ phase space (shown by the red line in Fig. 4d). These phases may be characterized by their opinion as :

- *weak consensus, mild opinion (“Mild”)*: There is not a single large cluster of agents holding *exactly* the same opinion within the precision defined in this work (see Methods), instead a large number of agents hold *neighbouring opinions* in the central region of the opinion space.
- *consensus, extreme opinion (“Skewed’)*: The opinion of the largest cluster lies far from the central region.
- *unanimity, extreme opinion (“U-turn”)*: The opinion of the largest cluster lies at one of the extremes and the largest cluster includes all the agents in the population.

In Fig. 5a we show the example of a society that evolves towards the mild phase. The final state is characterized by a broad strand where a large number of agents hold an opinion around $x=0.5$, however they are not close enough so as to form a unique cluster within the criteria used in this work to distinguish opinion differences (see Methods). This is the reason why the largest cluster size is low in the scatter plot, although the fraction of the population holding extremist opinions is comparatively small. Nevertheless, these extremists prevent the convergence of the central agents to a strong consensus.

Figure 5b shows the example of a society which ends up in the skewed phase, showing a majoritarian extreme opinion. As the initial opinions are uniformly distributed in the interval, there is a symmetry around the central one and a tendency to this central opinion is visible at the beginning of the dynamics, however at some point, agents with a central opinion are attracted to either of the extremes, and the agents that are near the other extreme stop interacting with the majoritarian strand. Nevertheless the extremist cluster contains a large majority of the society, hence the large values of *S* with an extremist opinion in the scatter plot.

Of special interest is the formation of the U-turn phase, shown in Fig. 5c. At an intermediate stage, the dynamics is similar to that of the skewed phase; however some open-minded agents in the majoritarian strand given their position in the network, are able to form a bridge between the strand and the isolated extremist agents on the opposite side of the opinion interval. This small set of agents is enough to completely overturn the majority of the society into the opposed extremist opinion.

**Figure 6:**
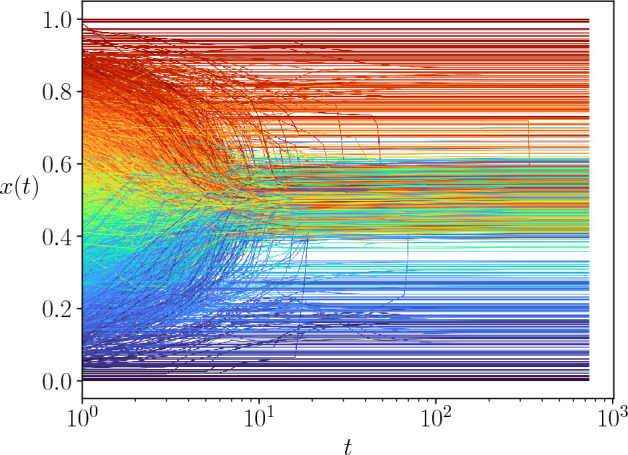
Example of the opinion evolution of the agents for a society where the interactions are constrained by an Erdős–Rényi network of $\langle k \rangle = 10$, with a large proportion of open-minded agents, $\varepsilon _l = 0.05$, $\varepsilon _u = 0.55$, $N=92681$. Note that for readability and computational cost, only the evolution of 4000 agents taken at random is displayed.

Both extremist societies are the result of the interplay of the agents’ heterogeneity with the network of potential interactions, and they are absent from both the heterogeneous mixed population case^16^ and the homogeneous model in networks^11^. It should be noticed that the situation where the whole society may suddenly change from one extreme of the opinion space to the other, does not require the presence of agents with very large confidence values, the existence of a few agents with $\varepsilon _u \approx 0.3$ is enough for this to occur. This phenomenon is observed in both types of studied random networks (see Supplementary Material, section III.C, for examples corresponding to the Barabási–Albert network).

Let us now analyse the behaviour of the system for large confidence values. At $\varepsilon _u >0.55$ the society contains many agents with large potential for compromising: as they have large confidence values, they interact with many of their neighbours. It may seem paradoxical then, that all the realizations have $S \longrightarrow 0$ in the scatter plot. Figure 6 shows that for large $\varepsilon _u$ the situation corresponds to the mild phase: open-minded agents rapidly integrate the central strand, which is pulled apart by the remaining closed-minded ones that have been left aside on both extremes of the opinion interval. Again, this situation requires both, agents heterogeneity and the interactions being locally constrained by a network.

**Figure 7:**
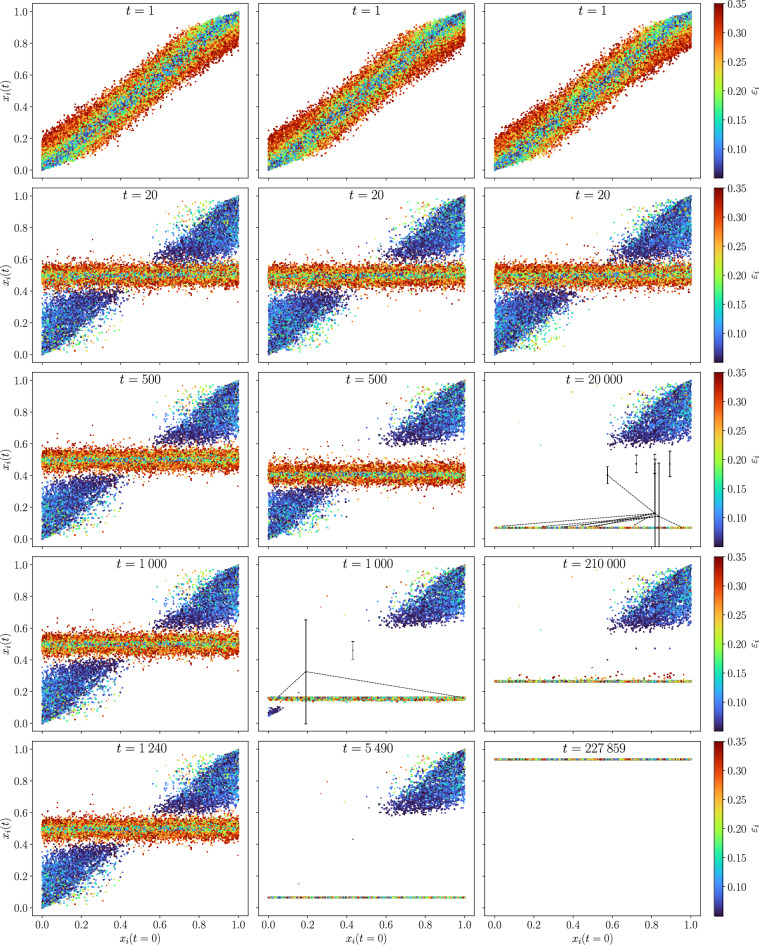
Snapshots of the evolution of the agents opinion as a function of their initial opinion, with the size of the dots proportional to the degree, color-coded by their confidence for a society of $N=92681$ agents where the interactions are constrained by an Erdős–Rényi network of $\langle k \rangle = 10$, and with confidence interval $\varepsilon _l = 0.05$, $\varepsilon _u = 0.35$. Rows correspond to snapshots taken at different times. Notice the variation in convergence times for the different phases, plotted in the last row. Left column: Mild phase. Middle column: Skewed phase. Right column: U-turn phase. Bridges are highlighted in some frames: solid black vertical lines represent the confidence interval of the agents and dotted lines represent *active links* (i.e. agents are topologically connected and within confidence reach).

In order to illustrate how these phases are formed, Fig. 7 shows snapshots of the opinion of the agents, $x_i(t)$, as a function of their initial opinion, $x_i (0)$, at the times marked by vertical lines on the trajectories of Fig. 5 (see video in the Supplementary Material, section III.B and III.C for complete evolution). The agents’ confidences, $\varepsilon _i$ are also indicated by the color scale.

In the three cases the central opinion region is quickly populated by agents with large confidences, while those with low confidences and extreme initial opinions remain with extreme opinions for longer times (second row of Fig. 7). In the left column, corresponding to the mild opinion phase, this situation remains stable and the central strand does not become dense because there are agents with extreme opinions and small confidences on both sides of it, which prevent the central ones from drifting towards either extreme. In the middle column, corresponding to the skewed phase, we observe the same initial dynamics, but the agents in one extreme succeed in pulling down the whole group of mild opinion because many of the open-minded agents within it are able to interact (and adopt) the extremist opinion while the extremist ones do not modify theirs. Finally the right column shows the mechanism responsible for the U-turn trajectories which leads to an unanimous extremist phase. First, a large fraction of the society is pulled towards one extreme of the opinion interval, as in the previous case, but there are open-minded agents who can act as a bridge with the close-minded extremist ones in the opposite extreme of the opinion space. The latter finally succeed in slowly pulling the whole society towards their opinion. The confidence interval of the open-minded agents who form the bridge are represented by the vertical lines of Fig. 7. These three dynamics have different time scales, the U-turn one being two order of magnitude longer that the others. The key ingredient that determines whether the dynamics will lead to a skewed or to a U-turn phase is the presence of *active* bridges. At $t=1000$ in the middle column of Fig. 7, we can see the potential for a bridge: there is a very open-minded agent that is slightly “left behind” from the main cluster and a close-minded agent that is within its confidence reach. However as there is no topological link between the two, the bridge cannot be “activated” and the phase remains skewed. On the contrary, at $t=20{,}000$ in the right column, the isolated open-minded agents and the close-minded agents that act as “anchor points”, are topologically connected and within confidence reach. The bridges are therefore active, and through repeated interactions, they will end up pulling the entire main cluster toward the other opposite extremist opinion.

**Figure 8:**
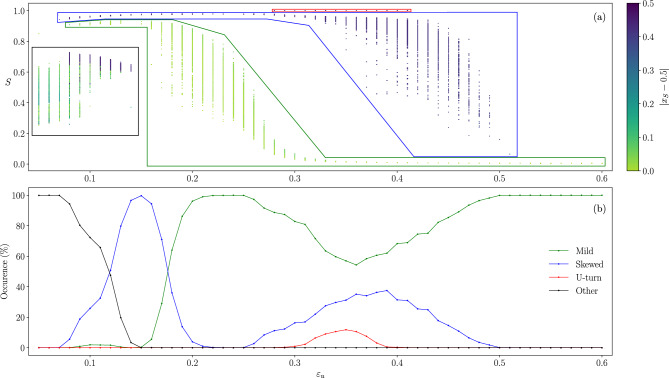
Probability of each type of phase for societies having $\varepsilon _l = 0.05$, as a function of $\varepsilon _u$, for $N=92681$ and interactions constrained by an Erdős–Rényi network of $\langle k \rangle = 10$. Averages calculated over 1000 samples. (**a**) The coloured boxes represent the samples used to compute the percentages shown in panel (**b**). (**b**) Probability of occurrence of each of the three phases observed in the steady state.

Finally, although one cannot predict for a single sample which will be its steady state, it is possible to estimate the probability of occurrence of each of the three phases. Figure 8 shows that it strongly depends on interval $[\varepsilon _l$ , $\varepsilon _u]$ characterizing the society. The probabilities have been calculated by computing, for $\varepsilon _l=0.05$ and each $\varepsilon _u$, the fractions of samples that end up in each of the three phases. To do so we consider a phase as “skewed” , when the opinion of the agents on the largest cluster is $|x_S-0.5| > 0.3$. The samples included in each case are shown by the coloured boxes of the scatter plot of Fig. 8a. The label “other” corresponds to the black box, where all agents of the society have very low confidences such that final state of the opinion remains in general very fragmented.

In particular, the probability of observing the society drastically changing from one extreme of the opinion space to the opposite one, requires the presence of agents who are prone to compromise with others holding rather different opinions, although not with *any* opinion. It should be noticed that the values of $\varepsilon _u$ at which the U-turn phase occurs are smaller than the trivial value $\varepsilon _u = 0.5$, which allows the interaction between the extreme opinions and the central one. In this region of the phase space the three phases may occur.

**Figure 9:**
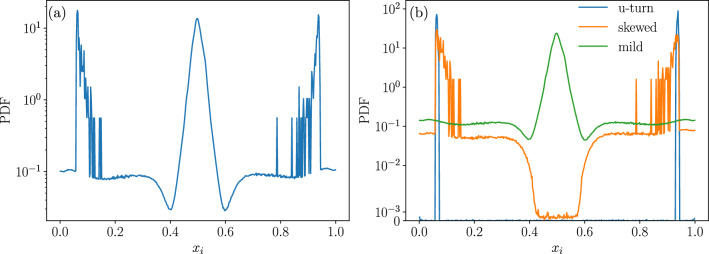
Distribution of the final opinions of all the agents in a society of size $N=92681$, where the interactions are constrained by an Erdős–Rényi network of $\langle k \rangle = 10$, and with confidence interval $\varepsilon _l = 0.05$, $\varepsilon _u = 0.35$, 1000 samples. (**a**) All realizations. (**b**) Realizations separated by the type of the final state: mild phase; skewed phase and U-turn phase.

The distribution of the final opinions for each of these phases is shown in Fig. 9 for the interval [0.05, 0.35]. The distribution has been computed considering the opinions of all the agents in the steady state (not only those corresponding to members of the largest cluster), for of all the realizations, thus confirming the results illustrated by single trajectories of Fig. 5.

### Particular case: lattices

**Figure 10:**
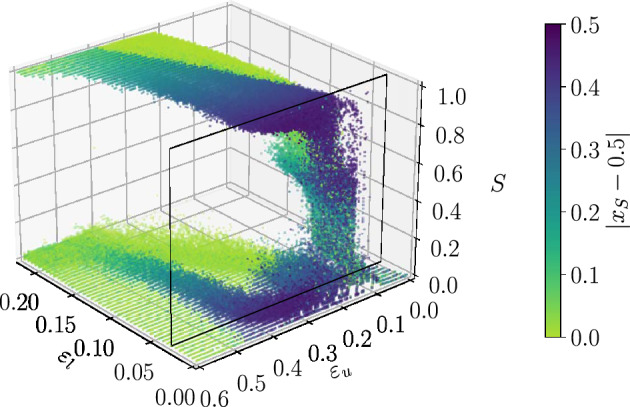
3D representation of the scatter plot of the sizes of the largest cluster in the steady state for a system of size $N=16384$, in a Square Lattice with up to third neighbour interactions. The color map indicates the extremism of the majoritarian cluster. The slice at $\varepsilon _l=0.03$ (vertical red line in Fig. 1) is outlined in black.

For the sake of comparison, we have also studied the case where the possible interactions are constrained by a lattice. The study of the homogeneous system has shown that the outcomes of the HK dynamics are qualitatively different in lattices and in random networks^11^. Figure 1 shows that this remains true for the heterogeneous model. Figure 10 shows the 3D scatter plot of the sizes of the largest clusters in the steady state, for a system of $N=16384$, in a square lattice with up to third neighbours interactions, which leads to a coordination number (degree) of $k = 12$, comparable to the average degree of the studied networks. The main difference with the latter is that, for lattices, the coexistent weak and strong consensus phases contain agents whose opinions may be either mild or extremist, while in networks we observe a clear separation of weak consensus around a mild opinion and strong consensus around extremist ones.

**Figure 11:**
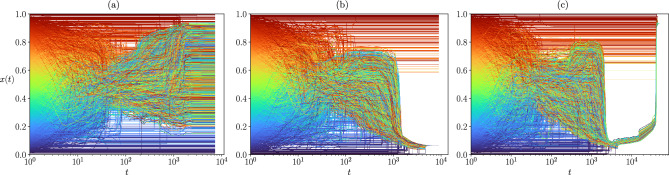
Example of trajectories for a system of size $N = 16384$, in a Square Lattice with up to third neighbour interaction, with $\varepsilon _l = 0.03$, $\varepsilon _u = 0.35$. Note that for readability and computational cost, only the evolution of 4000 agents taken at random is displayed. (**a**) Symmetric state around final opinions spanning a large central fraction of the opinion space, (**b**) Skewed extremist phase, (**c**) U-turn phase, notice the discrete jumps in opinion during the transient.

The evolution of the agents is also very different from that of random networks, as shown by the trajectories of Fig. 11, in particular because there is no initial tendency to a (weak) consensus around a mild opinion. Here, the trajectories occupy a very broad region of the opinion space.

Surprisingly, we have observed some “U-turn” trajectories. This is counter-intuitive because topological bridges do not exist in a lattice (all agents are connected to the same amount of neighbours). In fact, we found that the mechanism leading to the massive opinion change from one extreme of the opinion space to the other is different from that of networks. In networks, a few agents who have active links with two groups of opposite opinions, do not merge with any of them and succeed in bringing them together after a long transient. Here we observe successive jumps in opinion of large domains of agents. It should be noticed that lattices are embedded in a physical space, giving rise to the notion of neighbours of different order (nearest neighbours, second order neighbours, etc). In order to have comparable average degree, the results presented here correspond to a square lattice with up to third neighbours interactions. The lattice contains spatial domains of different opinions that at a given time of the evolution are delimited by inactive links. However, agents inside one opinion domain may establish an active link directly inside the other opinion domain, via second or third neighbours interactions, allowing for a further evolution that might overturn the global opinion state from one extreme to the other (see videos in section III.D of the Supplementary Material).

A particularity of lattices is the existence of some rare samples in the region of very low $\varepsilon _l$ which do not converge. In these samples, we observe a small, localized region of the lattice that enters in a cycle where the same group of neighbours keeps varying their opinion periodically. The confidence interval where such samples are found, is represented by a white pixel in the phase diagram of Fig. 1 to indicate that, due to this effect, we have not computed the average size of the largest cluster, $\langle S \rangle$. Randomness in the connections destroys this effect. 3D animations of the phase diagrams coded by the extremism of the final opinion, for all studied topologies, can be found in the Supplementary Material for a detailed comparison.

In order to compare with the random networks, we explored in detail different societies with $\varepsilon _l=0.03$ (vertical red line in Fig. 1), a region which shows similar $\langle S \rangle$ behaviour as the random networks as $\varepsilon _u$ increases. In this region typical trajectories are shown in Fig. 11.

## Discussion

In this work we study a bounded confidence model of opinion dynamics, which combines heterogeneity in the agents’ idiosyncratic properties, materialized by a quenched disorder, with interactions that are constrained by a network of social contacts. Our results show that, in presence of very close-minded agents, the existence of others that have a tendency to compromise with their neighbours holding an opinion quite different from theirs, may lead a large amount of the population that started to move to an intermediate opinion, to drift towards an extremist one. The drift to the extreme opinion in a mixed population HK model has been observed in Refs.^14,18^ for an unbalanced binary distribution of the confidences of the agents, with $20\%$ of the agents having $\varepsilon =0.3$, over the critical value of the homogeneous system, and the remaining $80\%$ having $\varepsilon =0.1$. However, when the agents’ confidences are distributed continuously over a interval $[\varepsilon _l,\varepsilon _u]$, this effect is not observed^16^. The drift to the extremes shown here is caused by the joint effect of the heterogeneous distribution of confidences and the constraining network of contacts.

More spectacularly, if some of the compromising agents find among their social contacts, another one which is in turn interacting with others holding a minoritarian opinion on the opposite extreme, the whole population may be overturned from one extreme to the other.

This result is relevant for applications in real settings which involve randomness in social ties and heterogeneity in the agents’ properties. It calls the attention about the usage of popular concepts as “compromise” and “open-mindedness”, which are usually endowed with a positive connotation, as they are considered leading properties which decrease conflict and promote consensus in society, avoiding extreme positions. Here we show how this tendency to compromise may not only lead the whole society to an extremist position but also to a sudden change from an extreme to the other of the opinion space. It is important to notice that this phenomenon occurs for societies with agents holding confidence values that are well below the trivial value of $\varepsilon = 0.5$, which would allow even the most extremist agents to interact with the middle opinion.

This phenomenon results from the interplay between the heterogeneity both in the agents’ confidences and in their connections, as it is observed neither in the heterogeneous model in the mixed population nor in the homogeneous model in networks. The key ingredient being the possibility to bridge among two different opinion clusters, where one of them contains open-minded agents and the other close-minded ones. The complete overturn of the society from an opinion to another requires a longer time scale. This is an issue of practical importance if one considers that, in real systems, the evolution time may be limited in such a way that the system can or cannot reach a steady state. This is typically the case of the time allowed to the population to form an opinion during electoral campaigns.

This article also questions the pertinence of a standard order parameter, $\langle S \rangle$, largely used in the study of opinion dynamics models^1^. It also recalls the importance of the size effects, which unfortunately are not sufficiently taken into account in the literature, where typical population sizes are around $N=1000$ and rarely go over $N=10{,}000$. Most of the findings shown here would have remained hidden had we limited our study to the behaviour of the standard order parameter for these typical sizes.

Our results allow us to statistically characterize the probability of observing a trajectory leading to a weak consensus around the mild opinion, to a strong consensus around one extreme opinion, or to an U-turn trajectory which overturns the society’s majoritarian opinion from one extreme to an unanimous agreement about opinion at the opposite extreme, according to the value of the confidences. Nevertheless, the question remains as to whether it would be possible to predict the final state from the properties of the agents, for instance, their confidence and position on the network. Unsurprisingly, a first machine learning test using a simple multi-layer perceptron architecture, did not lead to conclusive results, given that the number of total realisations (1000) is good for the statistical purposes, but remains small to train and test a machine learning algorithm. One important point is that the bridges, which here involve not only the position in the network and the confidence of the agent creating the bridge, but also its dynamical opinion value, occur during the evolution, therefore the details of the dynamics, and not only the quenched variables and initial conditions, intervene to create “U-turn” trajectories.

A next step along this line would be to introduce correlations between the agents’ properties and their social position, measured by their degree or their centrality. Would it then be possible to “tailor” the system so as to be able to drive the society’s opinion to a given region of the opinion space? This work is in progress.

## Conclusions

On the road to more realistic models of opinion dynamics, heterogeneity is an unavoidable issue. We see here how the simplest possible combination of heterogeneity -in the agents’ properties and in their interactions- leads to novel, almost paradoxical results. These results also raise the question about the attributes usually associated to individual attitudes: the tendency to compromise, abandoning one’s ideas or opinions is usually considered positively, arguing that it allows for a soft evolution to a consensus that will prevent the society from undergoing strong and radical changes. This works shows that this is not always the case, and that the final result depends on where the open and close-minded agents are placed in the network. This point is particularly relevant when one takes into account that nowadays, a large amount of the population builds its opinion on different subjects based on information circulating through different social networks. The links on the platforms giving access to these social networks are mediated by the algorithms which control to what information agents are exposed, coupling the heterogeneity of the connections with the dynamics of the opinion.

## Model and methods

### Hegselmann–Krause model

We study the Hegselmann–Krause model (HK), which describes a compromise dynamic under bounded confidence. Each agent *i*, $i = 1, N$, of the population is endowed with a dynamical continuous variable $x_i(t)\in [0,1]$ representing its *opinion*. In its heterogeneous version^14,16^ each agent is characterized by a quenched variable $\varepsilon _i$, modeling the *confidence* of the agent, i.e. its aptitude to interact with others holding different opinions. Every agent *i* may interact with any other agent *j* provided that their opinion differs in less than its confidence range $\varepsilon _i$, i.e. $x_j \in [x_i - \varepsilon _i, x_i + \varepsilon _i]$.

Here, we study the heterogeneous HK model, additionally constrained by the topology of an underlying static network as in Ref.^11^, that materializes the possible social contacts of the agents. Formally, we define the model on a graph $G = (V, E )$, where *V* is a set of vertices and *E* a set of non-directed edges, representing the agents and their interaction’s possibilities, respectively. The set of interaction partners of agent *i*, its *neighbourhood*, taking into account both the network constraint and the confidence $\varepsilon _i$, is therefore:

1 $$\begin{aligned} I(i, \overrightarrow{x} ) = \{1\le j \le N | |x_i - x_j |\le \varepsilon _i \wedge \{i,j\}\in E\}. \end{aligned}$$

The dynamics is defined in discrete time by a synchronous update of all agents at each time step. At each time step an agent *i* adopts the average opinion of all neighbours in the set defined by (1), i.e.

2 $$\begin{aligned} x_i(t+1) = \frac{1}{|I(i, \overrightarrow{x}(t))\vert } \sum _{j\in I(i, \overrightarrow{x}(t))} x_j(t). \end{aligned}$$

The networks of social ties are required to be connected simple graphs, i.e., no multi-edges or self-loops. Notice that, as in the original HK model, the active agent is explicitly included in the average of (2), which is required to prevent the synchronous update to induce artificial limit cycles in the dynamics.

For each run we start by drawing the initial opinions and the confidences from uniform distributions: $x_i(t=0) \in {\mathcal {U}}[0,1]$ and $\varepsilon _i \in {\mathcal {U}}[\varepsilon _l, \varepsilon _u]$, $\forall i=1,...,N$, with $\varepsilon _l \le \varepsilon _u$ and both $\varepsilon _l, \varepsilon _u \in [0,1]$.

The simulations are run by iterating (2) until the opinions (represented with 64-bits IEEE 754 floating-point numbers) converge, reaching a final *steady state*. The convergence criterion we use here requires that the sum of the changes over all agents is below a threshold, i.e.

3 $$\begin{aligned} \sum _{i=1}^{N} |x_i(t-1) - x_i(t) | < 10^{-4}. \end{aligned}$$

The main observable of interest is the relative size *S* of the largest cluster, a cluster being defined as the set of agents holding the same opinion within a tolerance of $10^{-4}$.

Unless stated otherwise, in the exploration of the full parameter space at each point $(\varepsilon _l, \varepsilon _u)$, results are computed by averaging over 100 realizations, while for the finite-size analysis at a given $\varepsilon _l$, averages are taken over 1000 independent realizations of the same society (same network type, and confidences $\varepsilon _i$ drawn from the same $[\varepsilon _l, \varepsilon _u]$ interval), each of which is called a *sample*.

Along with the average relative size of the largest cluster $\langle S \rangle$, we also compute its variance $\textrm{Var}\left( S\right)$ and the average convergence time $\langle T \rangle$. We also defined the *largest cluster size* entropy as:

4 $$\begin{aligned} \sigma = -\sum _{z\in Z} p_z \ln (p_z) \end{aligned}$$

where $p_z$ is the discrete probability for a given realization to present a largest cluster of size *z*, normalized to $\sum _{z\in Z}p_z = 1$, and *Z* is the set of all occurring largest cluster sizes among all the considered realizations. Note that this differs from the usual cluster size entropy, which would be defined by $p_s$, the probability for an arbitrary agent to be member of a cluster of relative size *s*. Our definition of the entropy will differentiate regions of the parameter space where the final state may present a broad or a sharp distribution of sizes, *S*, of the largest cluster of the different samples.

We introduce another metric, the *extremism*, *e*, defined as:

5 $$\begin{aligned} e = | x_S - 0.5 | \end{aligned}$$

where $x_S$ is the opinion held by the cluster of largest relative size *S*. As the opinion space is agnostic and symmetric with respect to the value $x=0.5$, we subtract 0.5 and take the absolute value. The extremism is thus defined $e \in [0,0.5]$ where $e=0$ denotes the majoritarian group having a totally *mild* opinion $x_S=0.5$, while $e=0.5$ means that the majoritarian group has an opinion at one extreme or the other of the opinion space: $x_S=0$ or $x_S=1$.

### Studied networks

We study a selection of network models chosen to cover different topological properties going from lattices to random networks, and from fully connected to scale-free networks. The networks considered here are^19^:

- *Fully connected network*: Agents arranged in a complete graph can be considered as not being restrained by any topology at all, as each agent is able, in principle, to interact with any other agent in the society. This is why it is also referred to as the mixed population society. This corresponds to the setup of the original HK model.
- *Square lattice (SL)*: We use periodic boundary conditions so as to ensure perfect regularity of the neighborhood of the agents. We vary the connectivity from nearest to third neighbours interactions, the latter giving $\langle k \rangle = k = 12$ which can be compared to the studied random networks with $\langle k \rangle =10$. Unlike other network topologies, lattices are embedded in a *D*-dimensional space. Here we concentrate our study on $D = 2$. Lattices show other important differences with respect to random networks: they have a long average path length and a large clustering coefficient.
- *c. Barabási–Albert (BA)*: This network model connects vertices using a preferential attachment procedure and leads to a scale-free degree distribution with a slope of $-3$ in the large-*N* limit . This growing network model starts with a clique of *m* nodes and adds sequentially new agents, each of which brings *m* new edges. Each of these edges is connected to the existing core with a probability proportional to the current degree of the target node. In this way nodes with a high degree gain more neighbors in a “the rich get richer” procedure. By construction, the networks of this ensemble have mean degree $\langle k\rangle = 2m$.
- *Erdős–Rényi (ER)*: Also known as uniform or binomial random graph, any two nodes are connected with probability *p*, leading to a network with uncorrelated connections and a mean degree $\langle k \rangle =Np$ , in the large-*N* limit. Here we study the sparse version for a fixed value of *Np* . For large *N* one needs to control that the generated networks are connected in order to avoid misleading results of the HK dynamics. Since we are conditioning on connectedness, the generation of ER realizations may not be trivial. Especially, it is known that almost all ER realizations are not connected if $N p < \ln (N)$, in the limit of large *N*. In our case, we only study $N p = 10$, which prohibits a rejection based sampling of connected ER realizations at $N \ge 16384$. However, the results for sizes up to $N = 92681$ [which is well above the threshold as $\ln (92681) \approx 11.4$, but apparently close enough that we encounter enough realizations of connected ER] already draw a convincing picture, such that we do not need to use more sophisticated methods, like Markov chain methods, to generate connected ER of larger sizes.

### Supplementary Information

Supplementary Information.

## Data Availability

The datasets generated during and/or analysed during the current study, as well as supplementary videos and 3D visualizations, are available at https://doi.org/10.5281/zenodo.7455640.
