# Supplementary material for: Phase coexistence in the fully heterogeneous Hegselmann–Krause opinion dynamics model
Source: Sci Rep. 2024 Jan 2;14:241. doi: 10.1038/s41598-023-50463-z (PMC10761677; doi:10.1038/s41598-023-50463-z)
Supplement: Supplementary file 1 — Supplementary Information. [file 41598_2023_50463_MOESM1_ESM.pdf]

Supplementary Material of ”Phase coexistence in the fully heterogeneous Hegselmann-Krause opinion dynamics model”

Rémi Perrier,[1,†] Hendrik Schawe,[1,‡] and Laura Hernández[1,*]

[1] *Laboratoire de Physique Théorique et Modélisation, UMR-8089 CNRS, CY Cergy Paris Université, France*

(Dated: October 31, 2023)

I. PHASE PLOTS

A. 3D visualisation

In this section we present videos that allow to inspect in 3D the scatter plots leading to the phase diagrams of Fig.1 of the main text, for different topologies. Each dot represents the normalized size of the largest opinion cluster at the steady state (on the Z-axis) for a given realization for a society where the confidences of the agents are drawn with uniform probability in the interval $[\varepsilon_l, \varepsilon_u]$ (X-axis and Y-axis respectively). The color code represents the level of extremism of the opinion of the largest cluster, x_S, given by $|x_S - 0.5|$ (green: $|x_S - 0.5| = 0$, blue: $|x_S - 0.5| = 0.5$).

- 360 rotation of 3D scatter plot for for a mixed population (fully connected network) space, $N = 16384$.
  scatter3D_el_eu_Smax_FC_N=16384.mp4

- 360 rotation of 3D scatter plot for a society constrained by an Erdös Rényi (ER) topology with $\langle k \rangle$=10, $N = 16384$.
  scatter3D_el_eu_Smax_ER_k=10_N=16384.mp4

- 360 rotation of 3D scatter plot for a society constrained by a Barabasi-Albert (BA) topology with $\langle k \rangle$=10, $N = 16384$.
  scatter3D_el_eu_Smax_BA_k=10_N=16384.mp4

- 360 rotation of 3D scatter plot for a society constrained by a Square Lattice (SL) topology with $\langle k \rangle = k$=4, 8, 12, $N = 16384$.
  scatter3D_el_eu_Smax_SL_k=4_N=16384.mp4
  scatter3D_el_eu_Smax_SL_k=8_N=16384.mp4
  scatter3D_el_eu_Smax_SL_k=12_N=16384.mp4

B. Lattices with different coordination number

Here we present, as a complement of Fig.1 of the main text, the phase diagrams for the square lattice topology where the interactions extend to first, second and third neighbours.

† remi.perrier@cyu.fr
‡ hendrik.schawe@cyu.fr
* laura.hernandez@cyu.fr

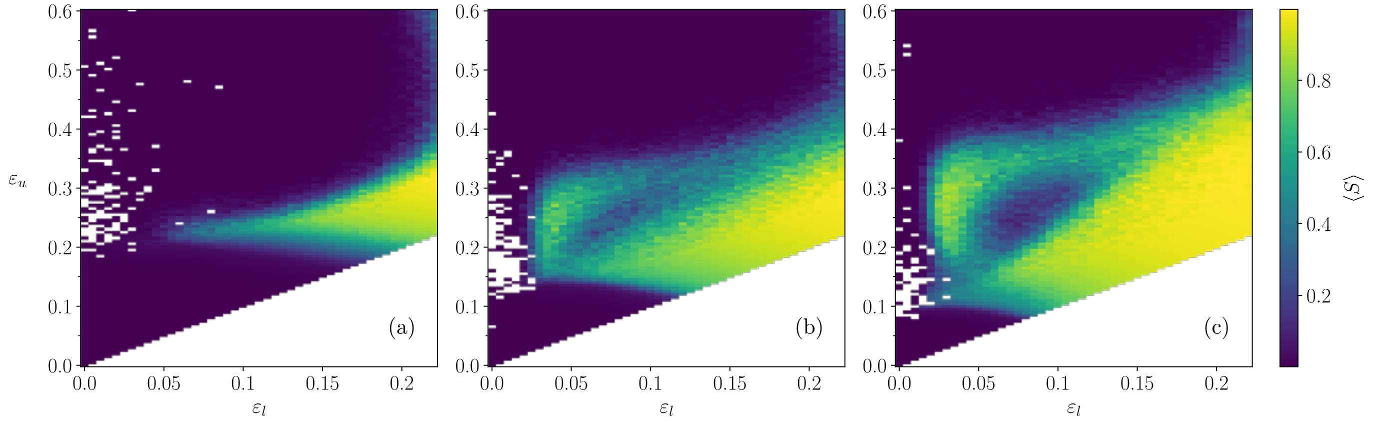

FIG. 1. Phase plots for a society where the interactions are limited by a square lattice, for a system with $N = 16384$ agents. The color map gives the average relative size of the largest cluster $\langle S\rangle$ averaged over 100 realizations. (a)Interactions limited to nearest neighbours in the lattice, $\langle k\rangle = k = 4$, (b)Interactions up to next nearest neighbours in the lattice, $\langle k\rangle = k = 8$, (c)Interactions up to third neighbours in the lattice, $\langle k\rangle = k = 12$. Note that some realizations did not converge in reasonable computing time according to the convergence criterion described in the Methods section of the main text. In order to avoid selection bias in the averaging, the corresponding points of the phase space are marked in white.

II. STUDY OF FINITE SIZE EFFECTS

A. 3D visualisation as a function of N

In this section we present videos that allow to inspect the scatter plots leading to Fig.2 of the main text, revealing the finite size effects, for different topologies in 3D. The Z-axis and the colormap are the same, representing the extremism.

- ER network with $\langle k\rangle$=10, $\varepsilon_l = 0.05$
  `scatter3D_el=0.05_eu_Smax_extremism_ER_k=10_SizeEffect.mp4`

- BA network with $\langle k\rangle$=10, $\varepsilon_l = 0.05$
  `scatter3D_el=0.05_eu_Smax_extremism_BA_k=10_SizeEffect.mp4`

B. 2D visualisation as a function of N

This section presents the same results as the previous section, but here visualized in 2D, for the values of confidence marked by a vertical line on Fig. 1 of the main text. Panels of Fig.4 in the main text are essentially still-frames from such a video.

- ER network with $\langle k\rangle$=10, $\varepsilon_l = 0.05$.
  `scatter2D_el=0.05_eu_Smax_extremism_ER_k=10_SizeEffect.mp4`

- BA network with $\langle k\rangle$=10,, $\varepsilon_l = 0.05$.
  `scatter2D_el=0.05_eu_Smax_extremism_BA_k=10_SizeEffect.mp4`

C. Other metrics

This section contains the results presented in Fig.2 along with additional metrics, namely: the average converging time according to the criterion explained in the section Methods of the main text; the variance of $< S >$; and the entropy of the size distributions of the largest clusters, S.

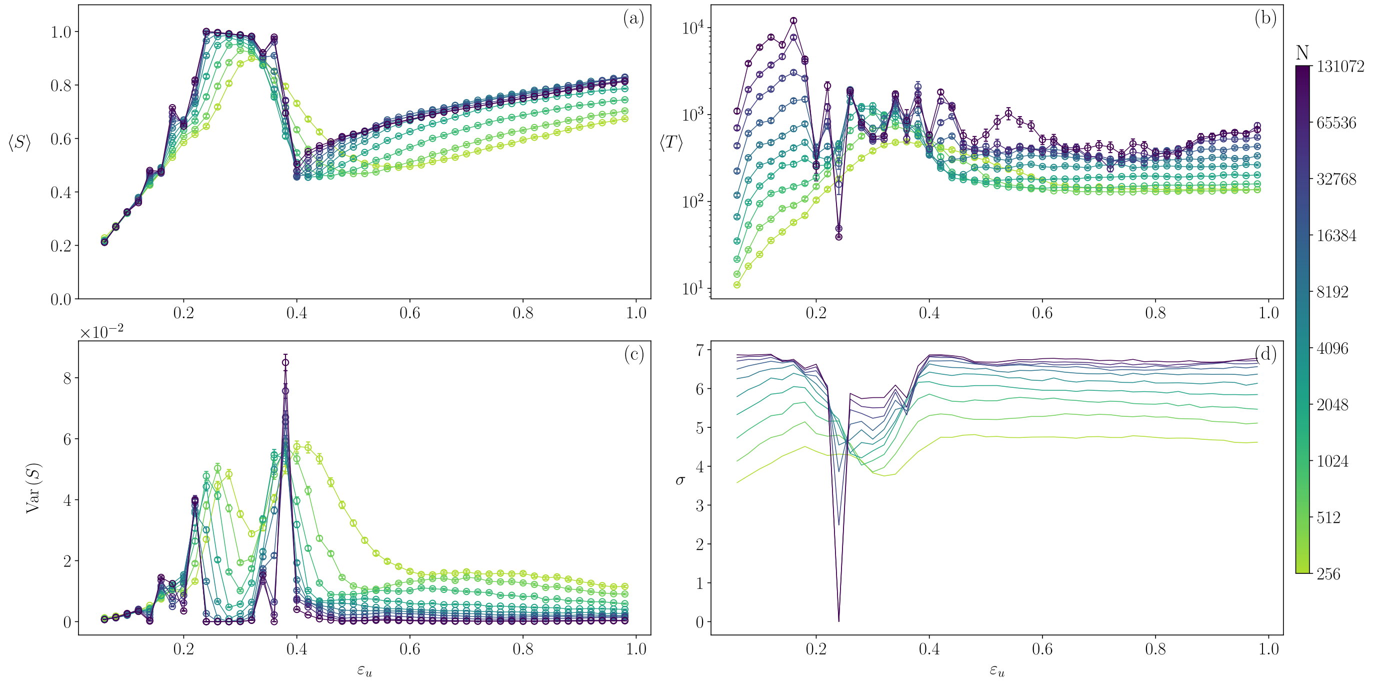

FIG. 2. Metrics for the fully connected network with $\langle k \rangle = 10$, $\varepsilon_l = 0.05$. Average over 1000 realizations. Data taken with the permission of the authors from [1]. (a) Average relative size of the largest cluster $\langle S \rangle$, (b) variance $\mathrm{Var}\,(S)$, (c) average convergence time $\langle T \rangle$, (d) largest cluster size entropy σ.

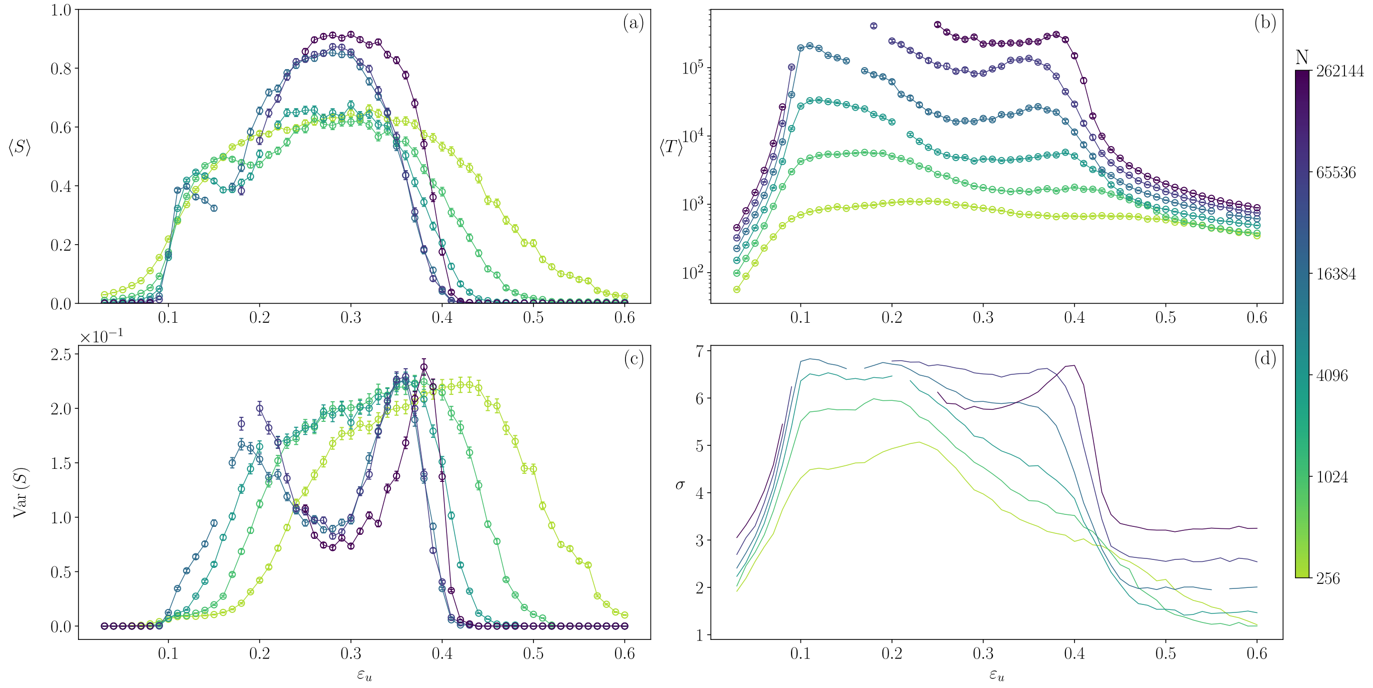

FIG. 3. Metrics for the square lattice including up to third neighbour interactions ($k = 12$), $\varepsilon_l = 0.03$. Average over 1000 realizations. (a) Average relative size of the largest cluster $\langle S \rangle$, (b) variance $\mathrm{Var}\,(S)$, (c) average convergence time $\langle T \rangle$, (d) largest cluster size entropy σ. Note that some realizations did not converge in reasonable computing time according to the convergence criterion described in the Methods section of the main text. In order to avoid selection bias in the averaging, the corresponding points are omitted.

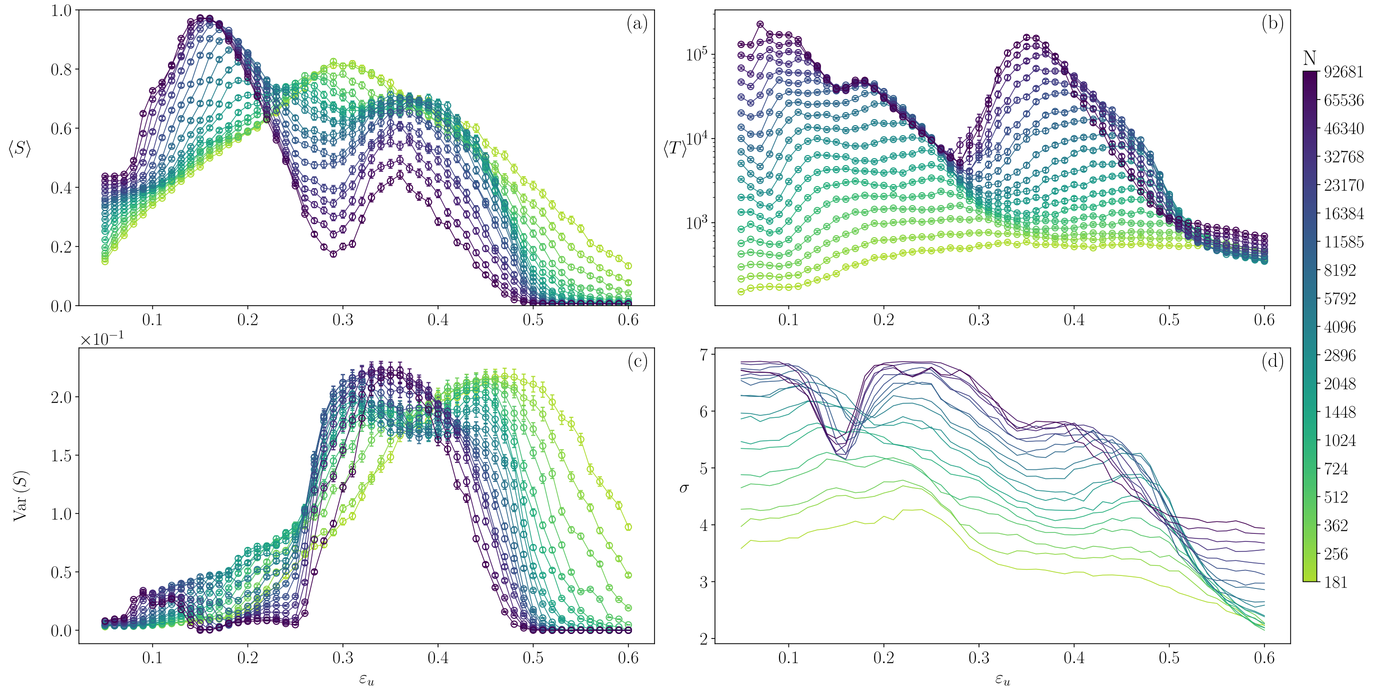

FIG. 4. Metrics for the Erdős–Rényi network with $\langle k\rangle = 10$, $\varepsilon_l = 0.05$. Average over 1000 realizations. (a) Average relative size of the largest cluster $\langle S\rangle$, (b) variance $\mathrm{Var}(S)$, (c) average convergence time $\langle T\rangle$, (d) largest cluster size entropy σ.

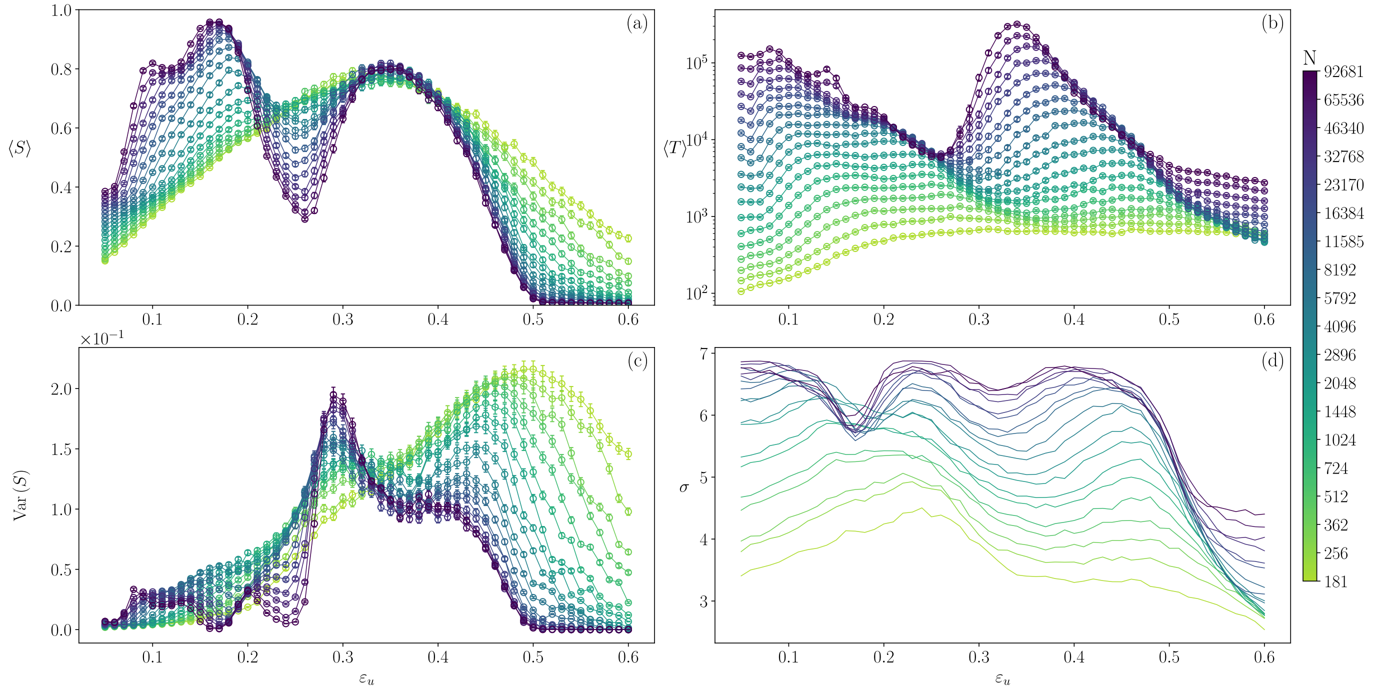

FIG. 5. Metrics for the Barabási-Albert network of $\langle k\rangle = 10$, $\varepsilon_l = 0.05$. Average over 1000 realizations. (a) Average relative size of the largest cluster $\langle S\rangle$, (b) variance $\mathrm{Var}(S)$, (c) average convergence time $\langle T\rangle$, (d) largest cluster size entropy σ.

III. PHASE COEXISTENCE

A. Effect of topology

This section presents the detail of size effects for the HK for the different topologies, for the largest system size available in each case.

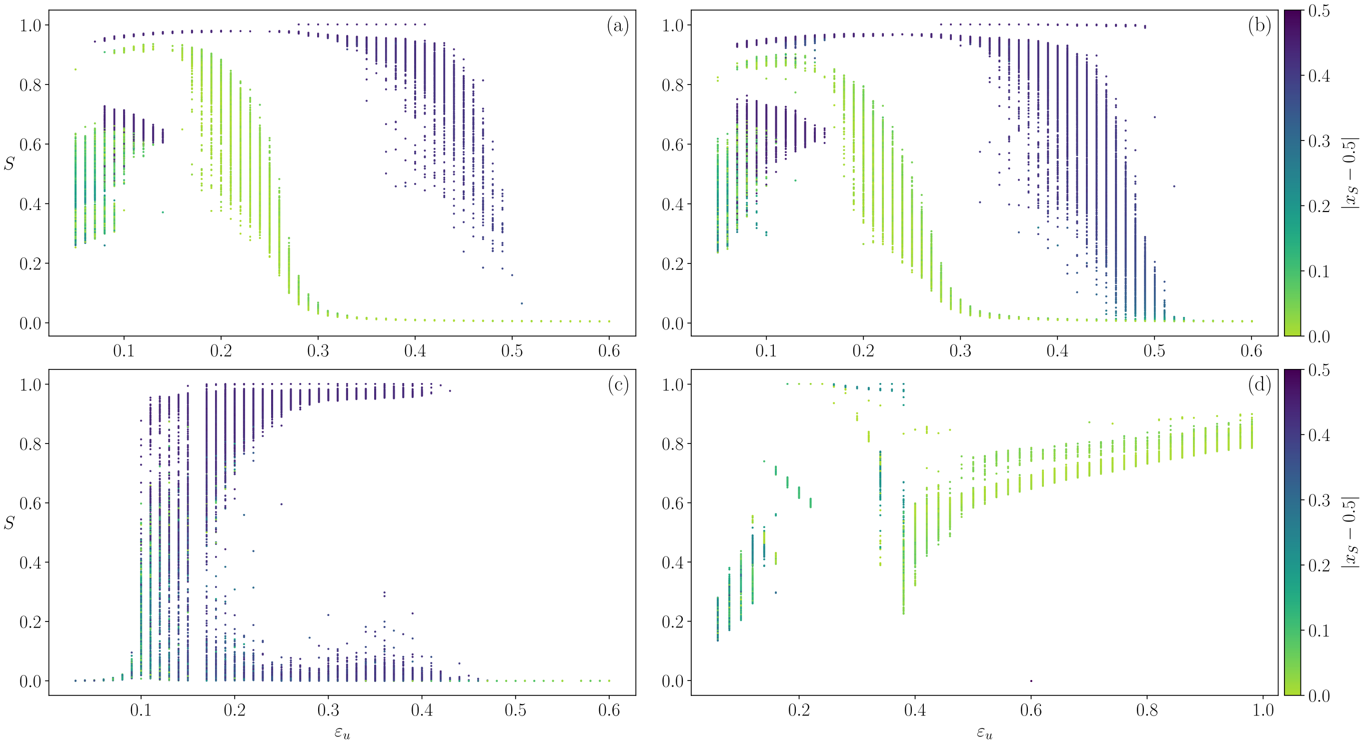

FIG. 6. (a): Erdős–Rényi network of $\langle k \rangle = 10$, $N = 92681$, $\varepsilon_l = 0.05$. (b): Barabási-Albert network of $\langle k \rangle = 10$, $N = 92681$, $\varepsilon_l = 0.05$. (c): Square lattice including up to third neighbour interactions, $N = 16384$, $\varepsilon_l = 0.03$. (d): Fully connected network (data taken with the permission of the authors from [1]), $\varepsilon_l = 0.05$. Note that due to the high occurrence of non-converging realizations at higher N, the scatter plot for the square lattice is not performed at the largest available size but at $N = 16384$.

B. Erdős-Rényi (ER)

This section presents the videos of the full evolution of $x_i(t)$ as a function of $x_i(0)$ leading to the snapshots of Fig.7 of the main text.

- ER network with $\langle k \rangle$=10, $\varepsilon_l = 0.05$, $\varepsilon_u = 0.35$, $N = 92681$. Weak consensus, mild opinion (*"Mild"*) phase. `scatter_x0_xt_ER_k=10_N=92681_mild.mp4`

- ER network with $\langle k \rangle$=10, $\varepsilon_l = 0.05$, $\varepsilon_u = 0.35$, $N = 92681$. Consensus extreme opinion (*"Skewed"*) phase. `scatter_x0_xt_ER_k=10_N=92681_skewed.mp4`

- ER network with $\langle k \rangle$=10, $\varepsilon_l = 0.05$, $\varepsilon_u = 0.35$, $N = 92681$. Unanimity extreme opinion (*"U-turn"*) phase. `scatter_x0_xt_ER_k=10_N=92681_uturn.mp4`

C. Barabási-Albert (BA)

This section presents the plots corresponding to Fig.5, Fig.7, and Fig.8 of the main text, for the Barabási-Albert network.

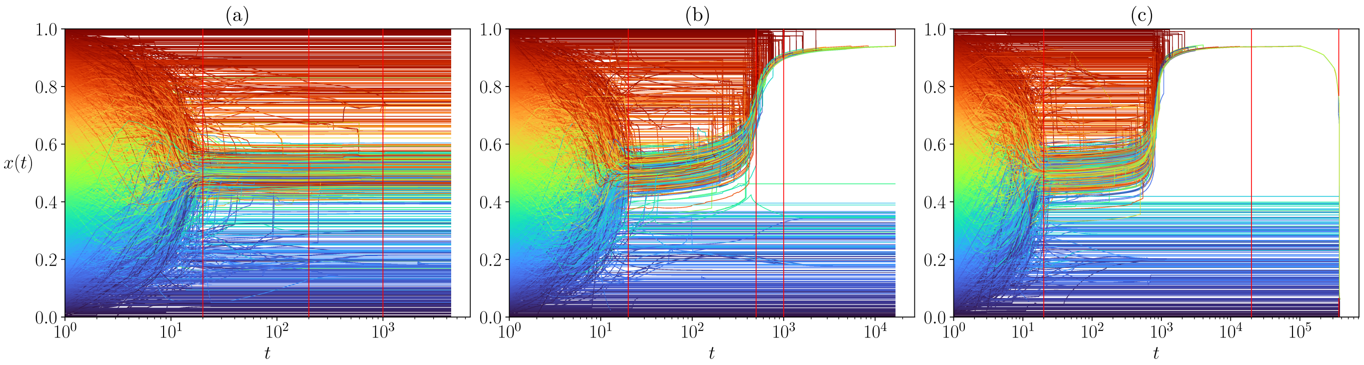

FIG. 7. Example of the opinion evolution of the agents for a society where the interactions are constrained by a Barabási-Albert network of $\langle k \rangle = 10$, and with confidence interval $\varepsilon_l = 0.05$, $\varepsilon_u = 0.35$, $N = 92681$. The vertical lines indicate the time at which snapshots shown in Fig. 9 were taken. (a) Mild opinion phase: The agents located around the central opinion do not meet the criterion ($\delta x < 10^{-3}$) to constitute a single cluster. (b) Skewed phase. The final state is a dense strand containing the majority of the society while a few agents remain isolated. (c) U-turn phase: The opinion of the society is overturned from one extreme to the opposite one, the whole society is involved. Note that for readability and computational cost, only a set of 4000 agents taken at random is displayed.

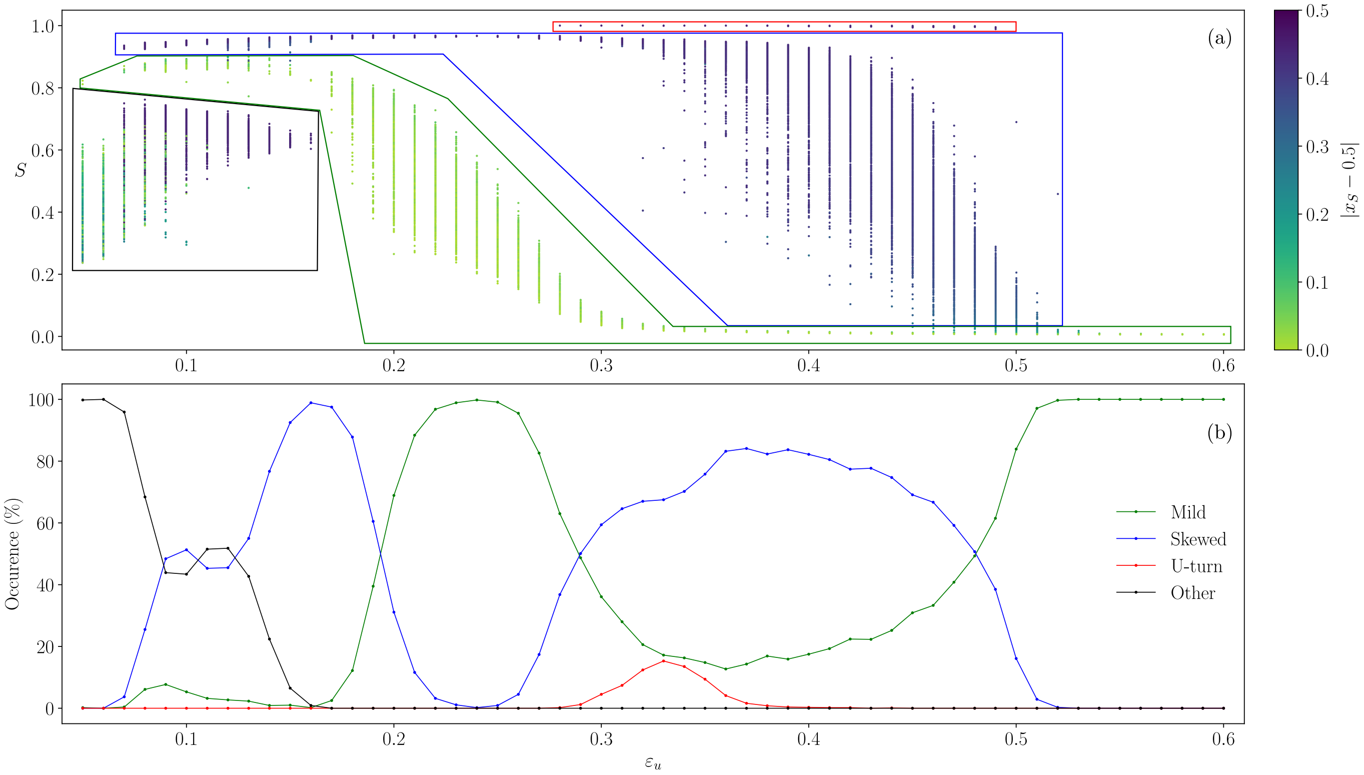

FIG. 8. Probability of each type of phase for societies having $\varepsilon_l = 0.05$, as a function of ε_u, for $N = 92681$ and interactions constrained by a Barabási-Albert network of $\langle k \rangle = 10$. Averages calculated over 1000 samples. (a) The coloured boxes represent the samples used to compute the percentages shown in panel(b). (b) probability of occurrence of each of the three phases observed in the steady state.

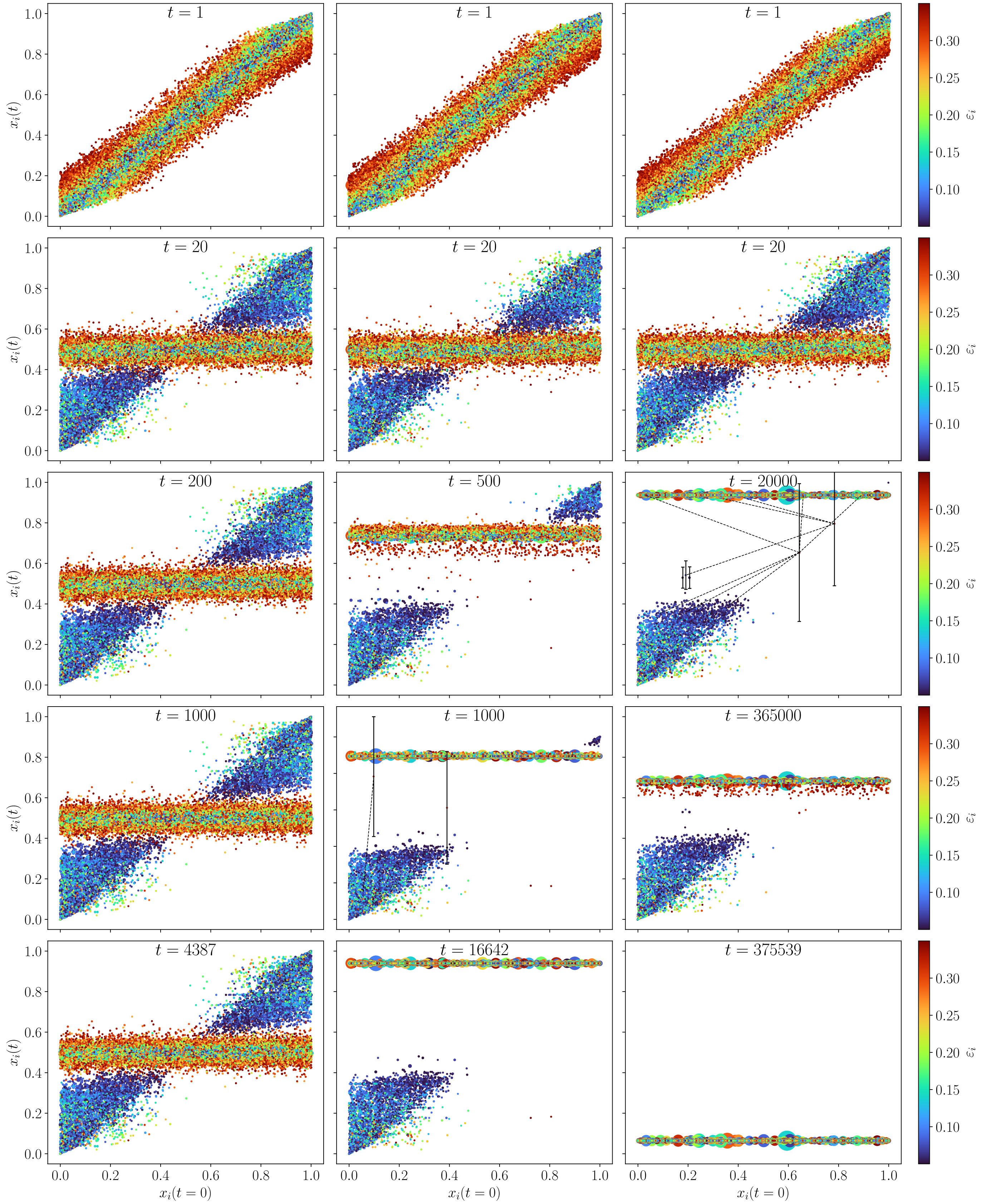

FIG. 9. Snapshots of the evolution of the agents opinion as a function of their initial opinion, with the size of the dots proportional to the degree, color-coded by their confidence for a society of $N = 92681$ agents where the interactions are constrained by a Barabási-Albert of $\langle k \rangle = 10$, and with confidence interval $\varepsilon_l = 0.05$, $\varepsilon_u = 0.35$. Notice that the time to final convergence is increasing from left to right. Left column: Mmild opinion phase. Middle column: Skewed phase. Right column: U-turn phase . Bridges are highlighted in some frames: solid black vertical lines represent the confidence interval of the agents and dotted lines represent *active links* (i.e. agents are topologically connected and within confidence reach).

The videos of the full evolution of agents opinion as a function of their initial opinion, the snapshots of Fig. 9 are taken from them:

- BA network with $\langle k \rangle$=10, $\varepsilon_l = 0.05$, $\varepsilon_u = 0.35$, $N = 92681$. Weak consensus, mild opinion (*"Mild"*) phase.
  scatter_x0_xt_BA_k=10_N=92681_mild.mp4

- BA network with $\langle k \rangle$=10, $\varepsilon_l = 0.05$, $\varepsilon_u = 0.35$, $N = 92681$. Consensus extreme opinion (*"Skewed"*) phase.
  scatter_x0_xt_BA_k=10_N=92681_skewed.mp4

- BA network with $\langle k \rangle$=10, $\varepsilon_l = 0.05$, $\varepsilon_u = 0.35$, $N = 92681$. Unanimity extreme opinion (*"U-turn"*) phase.
  scatter_x0_xt_BA_k=10_N=92681_uturn.mp4

D. Square Lattice with third degree neighbours

This section presents the videos for the trajectories shown in Fig.11 in the main text, for the Square Lattice with third degree neighbours. Because of the spatial embedding, these trajectories can be visualized in 2D.

- Square Lattice with $\langle k \rangle = k = 12$, $\varepsilon_l = 0.03$, $\varepsilon_u = 0.35$, $N = 163884$. Weak consensus, mild opinion (*"Mild"*) phase.
  traj_2D_SL_k=12_N=16384_mild.mp4

- Square Lattice with $\langle k \rangle = k = 12$, $\varepsilon_l = 0.03$, $\varepsilon_u = 0.35$, $N = 163884$. Consensus extreme opinion (*"Skewed"*) phase.
  traj_2D_SL_k=12_N=16384_skewed.mp4

- Square Lattice with $\langle k \rangle = k = 12$, $\varepsilon_l = 0.03$, $\varepsilon_u = 0.38$, $N = 163884$. Unanimity extreme opinion (*"U-turn"*) phase.
  traj_2D_SL_k=12_N=16384_uturn.mp4

[1] H. Schawe and L. Hernández, When open mindedness hinders consensus, Scientific reports **10**, 8273 (2020).
